# A new player in the dermis

**DOI:** 10.7554/eLife.68979

**Published:** 2021-05-10

**Authors:** Gyohei Egawa, Kenji Kabashima

**Affiliations:** 1 Department of Dermatology, Kyoto University Graduate School of MedicineKyotoJapan; 2 Singapore Immunology Network and Skin Research Institute of Singapore, Agency for Science, Technology and ResearchSingaporeSingapore

**Keywords:** skin, cell migration, dermis, lymph node, dendritic cell, Langerhans cell, Mouse

## Abstract

Langerhans-like cells located in the dermis can travel to lymph nodes where they modulate immune responses.

**Related research article** Sheng J, Chen Q, Wu X, Dong YW, Mayer J, Zhang J, Wang L, Bai X, Liang T, Sung YH, Goh WWB, Ronchese F, Ruedl C. 2021. Fate mapping analysis reveals a novel murine dermal migratory Langerhans-like cell population. *eLife* **10**:e65412. doi: 10.7554/eLife.65412

The skin is part of the first line of defense protecting the body from infection. It harbors a range of immune cells, including Langerhans cells (Langerhans, 1868). Initially thought to be part of the nervous system, Langerhans cells play an important role in the defense against pathogens (Schuler and Steinman, 1985).

Due to their characteristic, branch-like morphology, Langerhans cells are considered a subset of dendritic cells, which are bone-marrow derived leucocytes. As such, Langerhans cells have been thought to play an important role in detecting and transporting antigens (signature molecules from pathogens) to the lymph nodes and presenting them to other immune cells such as naïve T cells to initiate an immune response (Kubo et al., 2009).

However, it has been shown that enhanced antigen-specific immune responses in the skin can occur even in the absence of Langerhans cells (Bennett et al., 2005). Moreover, recent cell-fate mapping studies and gene expression analyses have revealed that Langerhans cells derive from the embryo rather than the bone marrow and have properties closer to macrophages than dendritic cells. Dubbed ‘macrophages in dendritic clothing’, the migratory capacity of Langerhans cells to travel from the epidermis to skin-draining lymph nodes has, however, been considered exceptional, especially since macrophages do not leave the tissues they reside in (Doebel et al., 2017).

Now, in eLife, Christiane Ruedl and colleagues at the Zhejiang University School of Medicine, the Nanyang Technological University and the Malaghan Institute of Medical Research – including Jianpeng Sheng as first author – report a new paradigm of Langerhans cells (Sheng et al., 2021). Sheng et al. tracked immune cells in the skin of mice and discovered that unlike other dendritic cells, Langerhans cells do not migrate to the lymph nodes.

Previously, dendritic cells in the skin have been classified into Langerhans cells, which reside in the epidermis, and three other types dendritic cells located in the dermis (Figure 1; Malissen et al., 2014). Sheng et al. have found a fourth population of dendritic cells in the dermis, which turned out to look a lot like Langerhans cells, and named them Langerhans-like cells. These lookalikes shared many signature genes with Langerhans cells.

**Figure 1.**
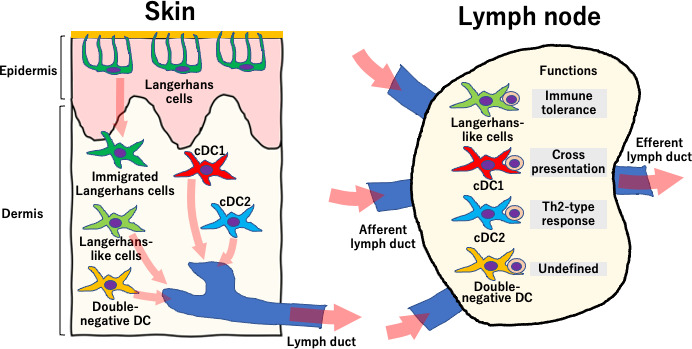
The roles of dendritic cells in the skin and the lymph nodes. The skin (left) is an important barrier to pathogens and contains Langerhans cells and four types of dermal dendritic cells. Langerhans cells (dark green) reside in the epidermis (dark green), although they can occasionally migrate into the dermis. The other four types of cells reside in the dermis and consist of Langerhans-like cells (light green), double-negative dendritic cells (yellow), and conventional dendritic cells 1 and 2 (cDC1 and cDC2, shown in red and blue respectively). The four cell types that reside in the dermis can travel through the lymphatic ducts into the skin-draining lymph nodes (right), where they interact with naïve T cells. Langerhans-like cells perform tasks related to immune tolerance, cDC1 cells do cross-presentation of antigens and cDC2 cells are involved in the Th2-type response to fight extracellular parasites and bacterial infection. Double-negative dendritic cells can also travel to the lymph nodes but their role there is not well defined.

Fate-mapping analyses of the bone marrow of mice confirmed that similar to Langerhans cells, Langerhans-like cells were resistant to radiation (which most immune cells are not). However, the lookalikes were gradually replaced by adult bone-marrow cells, suggesting that Langerhans-like cells are derived both from embryonic and adult bone marrow, while Langerhans cells are of embryonic origin.

Notably, Sheng et al. found only Langerhans-like cells, but not Langerhans cells, in the skin-draining lymph nodes, suggesting it is the lookalike cells that are able to travel. Moreover, using transgenic mice that can deplete Langerhans cells, Sheng et al. demonstrated that the lookalikes were found both in the skin and lymph nodes. This indicates that Langerhans cells and lookalike cells are indeed independent cell populations.

Previous research has shown that depending on the circumstances, Langerhans cells may be involved both in inducing dermatitis or reducing inflammation in the skin (Rajesh et al., 2019; Otsuka et al., 2018; Honda et al., 2019). Consistent with previous research, Sheng et al. demonstrated that removing Langerhans cells prolonged skin inflammation against hapten, a small molecule that can cause contact dermatitis (Kaplan et al., 2005). On the other hand, only Langerhans-like cells were able to develop an immune tolerance (that is, unresponsiveness) towards hapten. This may be relevant for maintaining an immunological tolerance to harmless substances in the absence of any obvious inflammation and help maintain homeostasis of the skin. These results indicate that Langerhans cells and Langerhans-like cells have distinct roles in skin immunity.

The finding that epidermal Langerhans cells do not appear to travel to skin-draining lymph nodes comes as a surprise and raises several questions. While Sheng et al. also identified ‘immigrated Langerhans cells’ in the dermis, it remains unclear if they play any role in this location and whether this is indeed their last destination. Also, if Langerhans cells do not migrate to the lymph nodes, how do they pass antigens to other dendritic cells in the skin?

Characterizing the distinct role of each dendritic cell population in the skin has long been one of the central goals of skin immunology. It will be interesting to see if the findings of Sheng et al. also apply to human skin and whether any counterparts of Langerhans-like cells can be identified there. A better understanding of the different roles of dendritic cells in the skin would certainly be beneficial for the development of treatments for various inflammatory skin disorders.
